# Supplementary material for: Slippery damper of an overlay for arresting and manipulating droplets on nonwetting surfaces
Source: Nat Commun. 2021 May 26;12:3154. doi: 10.1038/s41467-021-23511-3 (PMC8154893; doi:10.1038/s41467-021-23511-3)
Supplement: Supplementary file 1 — Supplementary Information [file 41467_2021_23511_MOESM1_ESM.pdf]

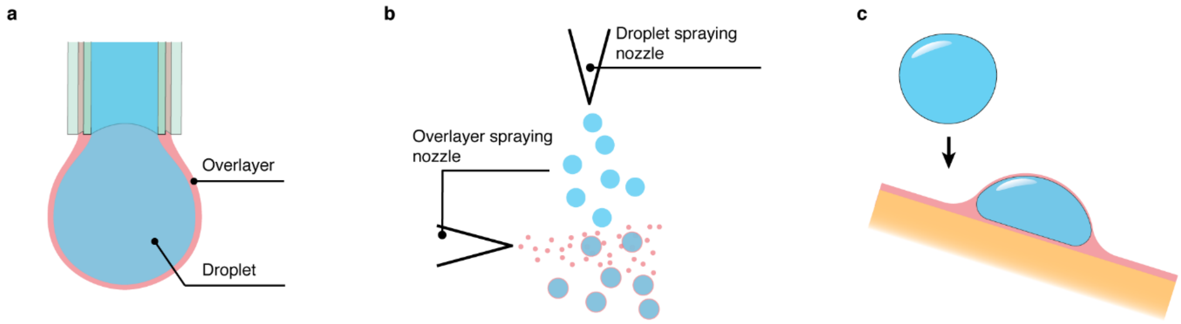

Supplementary Fig. 1. Overlaying methods. (a) A co-flow microfluidics device. (b) Counter spraying. (c) Sliding on the overlayer-liquid-infused surface.

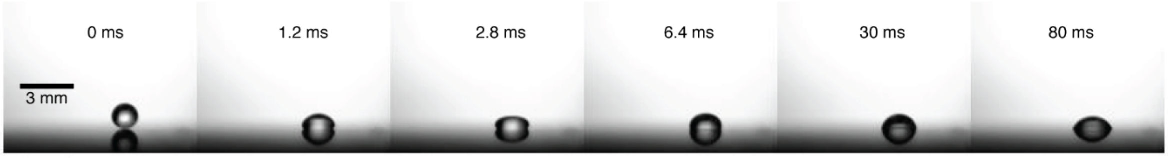

Supplementary Fig. 2. Impact of pure silicone oil. Sequential side view images showing the impact of a silicone oil droplet on a superhydrophobic surface. We = 15.5, viscosity of silicone oil is 4.6 mPa s.

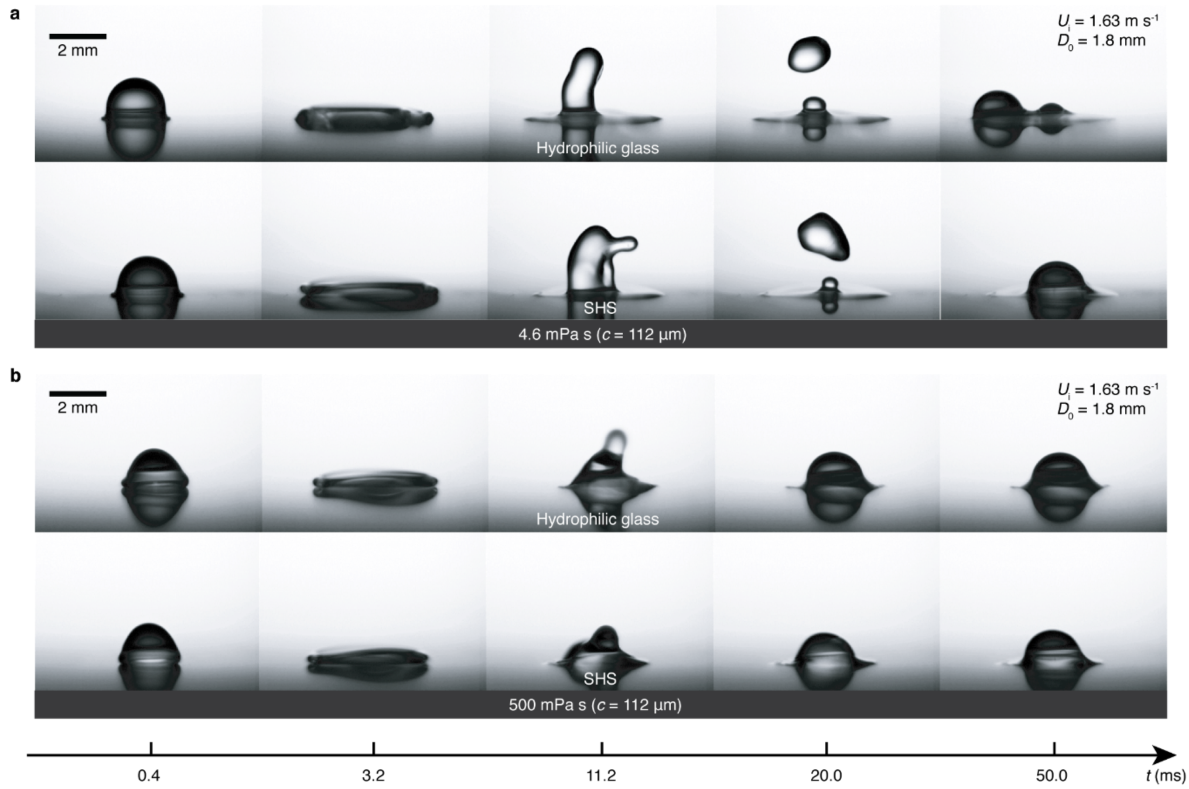

Supplementary Fig. 3. Overlayer viscosity. Sequential side view images contrast the impact outcome of water droplet overlaid with silicone oil of a viscosity of (a) 4.6 mPa s and (b) 500 mPa s on hydrophilic and superhydrophobic glass. Water core in 4.6-mPa s overlayer rebounds through pinch-off whereas that in 500-mPa s overlayer is arrested without rebound. At relatively low velocity (U_i = 1.63 m s^{-1}), impacting outcome is invariant, regardless of distinct substrate wettability.

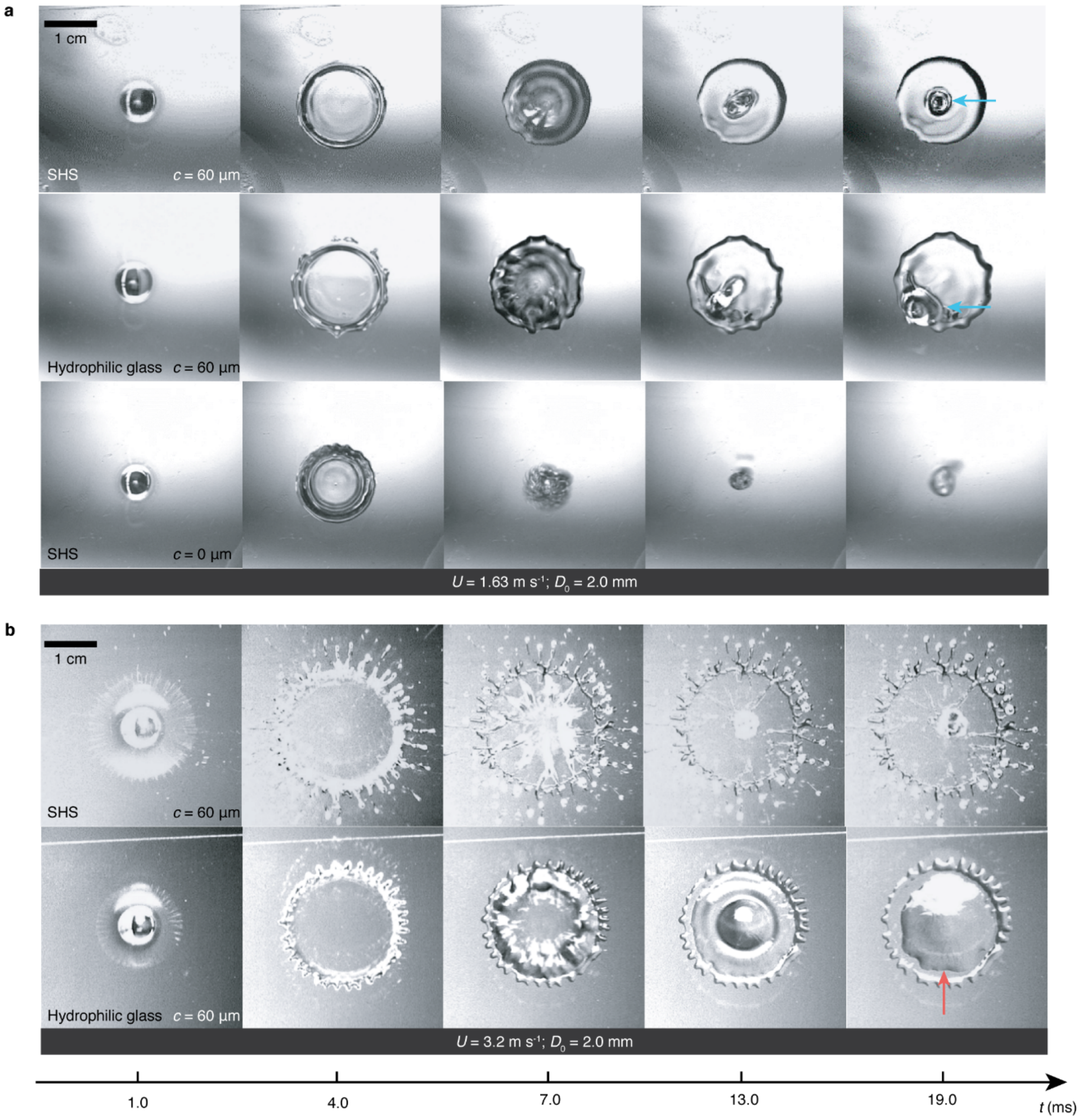

Supplementary Fig. 4. Subjacent lubricant film. Sequential bottom view images contrast the state of lubricant film for droplet impacting at a velocity of (a) 1.63 m s^{-1} and (b) 3.2 m s^{-1} on hydrophilic and superhydrophobic glass. At 1.63 m s^{-1}, lubricant film remains intact on both hydrophilic and superhydrophobic glass as water core slides atop the film (denoted by blue arrow). At 3.2 m s^{-1}, compared with that on superhydrophobic glass, the lubricant film breaks up on hydrophilic glass as water/glass contact line (denoted by red arrow) can be clearly observed.

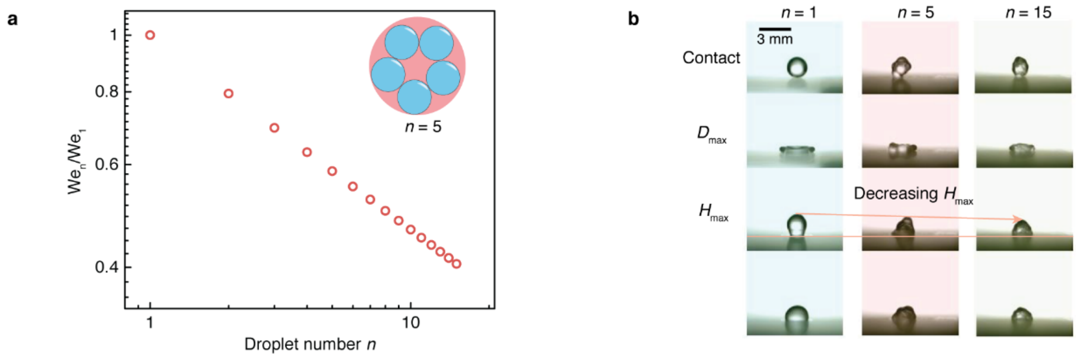

Supplementary Fig. 5. Overlaid multi-droplets. (a) By fixing the total droplet volume, the We number of individual core decreases as the droplet number increases. Insets show the schematic of an overlaid droplet containing five cores. (b) Droplets containing higher core number n rebound with lower H_{max}. The number n and the size of droplet are precisely tuned using the microfluidic device[1].

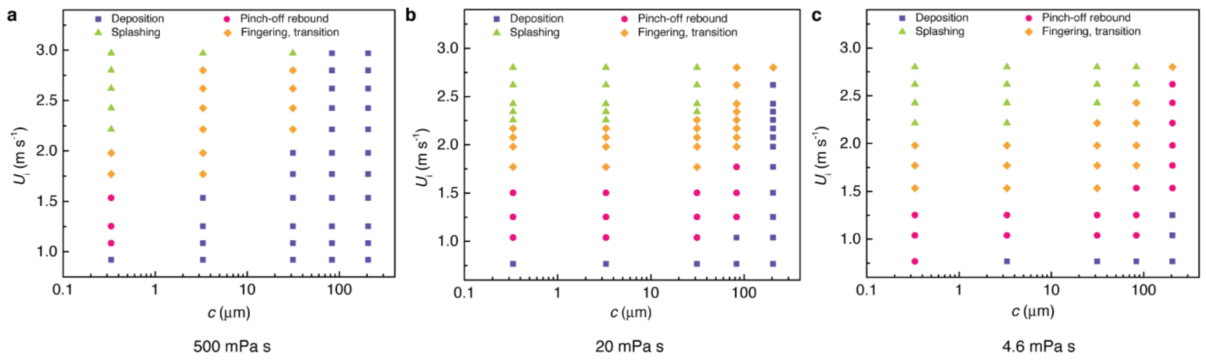

Supplementary Fig. 6. Phase diagram of impacting outcome. Phase diagram of water droplet overlaid with (a) 500-mPa s, (b) 20-mPa s, and (c) 4.6-mPa s silicone oil. Markers show experimental outcomes after impact (purple squares: deposition, magenta circles: pinch-off rebound, orange diamonds: fingering, transition, and green triangles: splashing) as a function of impacting velocity and overlayer thickness.

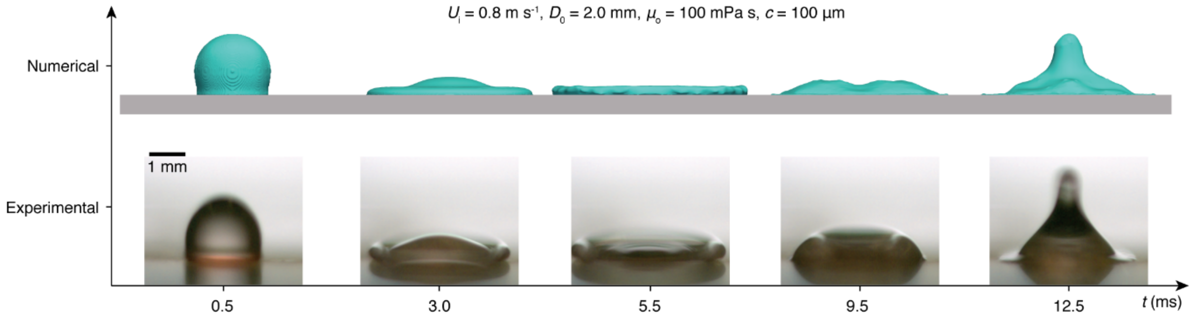

Supplementary Fig. 7. Numerically studied impacts. Sequential side view images showing the numerical and experimental shape evolution of an impacting silicone-oil-overlaid water droplet. The numerical results (top) match the experimental ones (down).

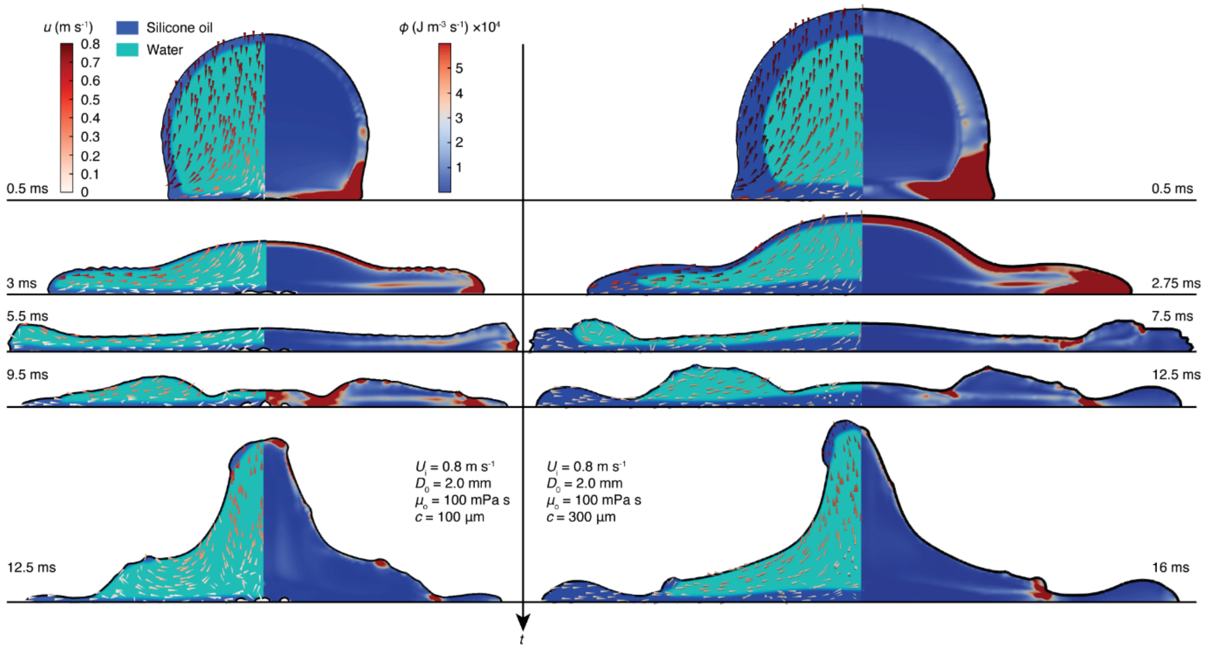

Supplementary Fig. 8. Overlayer thickness. Comparison of numerically calculated phase, flow field, and distribution of viscous dissipation rate of impacting droplets coated with 100-μm (left) and 300-μm (right) overlayer. Compared with the droplet of 100-μm-thick overlayer, receding of the droplet of 300-μm-thick overlayer is slightly slower but has similar H_{max}. The distribution of viscous dissipation rate is similar for both droplets. As thicker overlayer has extra amount of kinetic energy (same D_0), the viscous dissipation region is clearly larger for the droplet of 300-μm-thick overlayer.

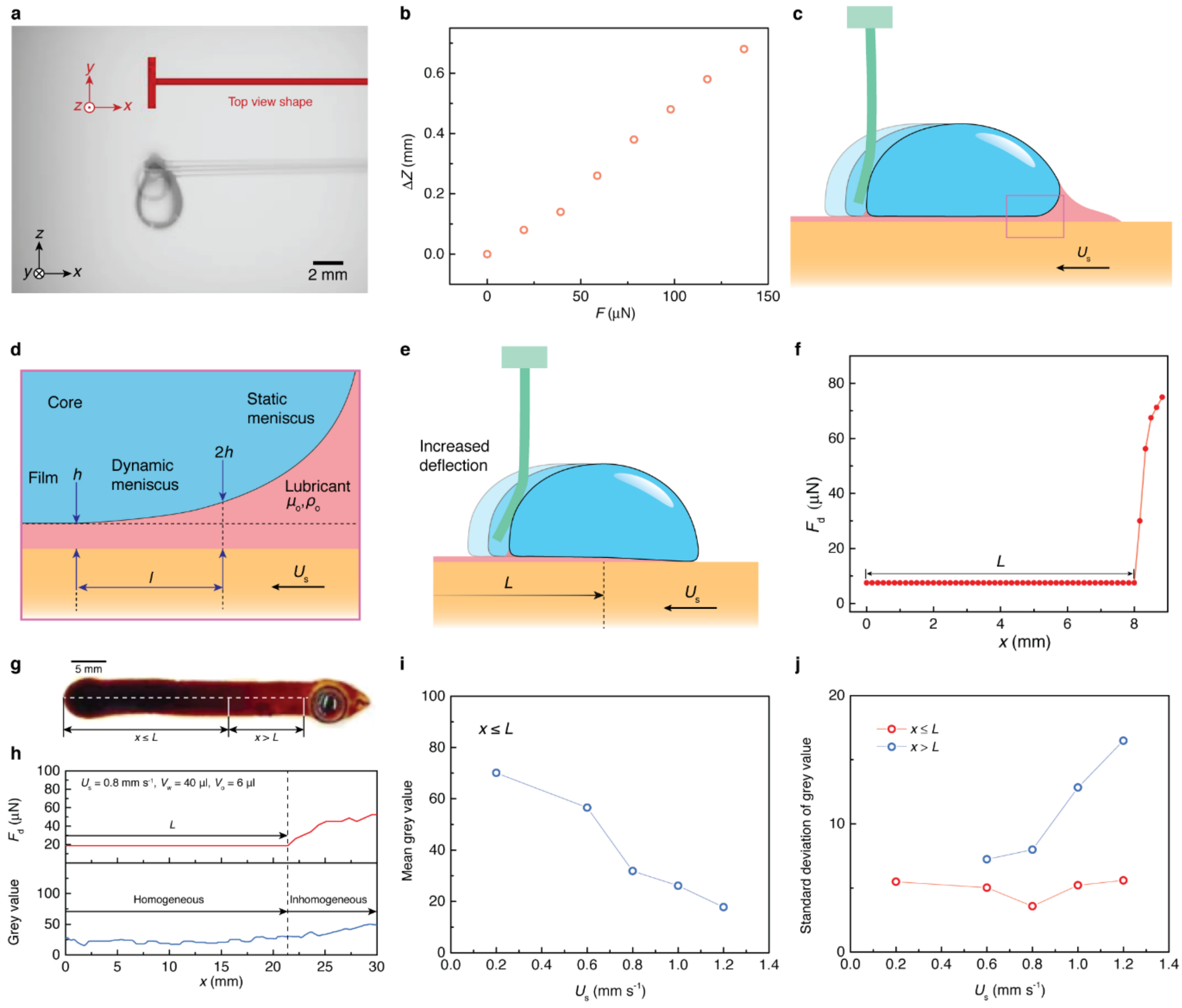

Supplementary Fig. 9. Roller support sliding. (a) Microscope side view image showing the structure of the cantilever force sensor. Droplets of varied weights are hang on tip of the cantilever (9 cm in length) to establish a relationship between the force F and deflection Δz. Inset is a top view schematic of the cantilever whose tip is modified to increase the contact area with the droplet to avoid ready droplet falloff. (b) Calibration of the force sensor. The deflection Δz increases with the acting force. Schematics showing (c) lubricated sliding of an overlaid droplet, (d) dynamic meniscus of sliding droplet front in (c), and (e) termination of lubricated sliding because of oil depletion. (f) Resistant force F_d probed using the force sensor during droplet sliding. F_d ramps as oil is insufficient to sustain the lubrication. The lubricated sliding distance L is obtained by real-time force measuring. (g) Top view image showing the droplet trail (sliding velocity $U_s = 0.8$ mm s^{-1}). Oil is dyed black to aid visualization. (h) Real-time F_d

and grey value of droplet trail along white dashed scan line in (g). For $x \leq L$, the grey value only slightly fluctuates, suggesting relatively constant lubricant thickness. For $x > L$, the grey value increases, suggesting decreasing lubricant thickness. (i) For $x \leq L$, the lubricant thickness (mean grey value) relates to the sliding velocity. (j) For different sliding velocities, the lubricant trail for $x \leq L$ is more homogeneous than that for $x > L$. Homogeneity is represented by the standard deviation of grey value.

Supplementary Note 1. Numerical study of impact.

The MultiphaseinterFoam solver on the OpenFOAM platfom is used to numerically study the impact of overlaid droplets[2]. Equations governing the dynamics of three immiscible and incompressible phase (air, silicone oil, and water) flows are as follows:

$$\nabla \cdot \mathbf{u} = 0 \tag{1}$$

$$\frac{\partial(\rho\mathbf{u})}{\partial t} + \nabla \cdot (\rho\mathbf{u}\mathbf{u}) = -\nabla p + \nabla\mu\left[\nabla\mathbf{u} + (\nabla\mathbf{u})^{\mathrm{T}}\right] + \rho\mathbf{g} + \mathbf{F}_{\mathrm{st}} \tag{2}$$

where p is pressure, $\mathbf{g}$ is gravitational acceleration, and $\mathbf{F}_{\mathrm{st}}$ is surface tension force.

Fluidic interfaces are described as follows:

$$\sum_{\mathrm{k}} \alpha_{\mathrm{k}} = 1 \text{ with k = 1, 2, 3, denoting air, water, and oil} \tag{3}$$

$$\frac{\partial \alpha_{\mathrm{k}}}{\partial t} + \nabla \cdot (\mathbf{u}\alpha_{\mathrm{k}}) + \nabla \cdot (\mathbf{u}_{\mathrm{r}}\alpha_{\mathrm{k}}(1-\alpha_{\mathrm{k}})) = 0 \tag{4}$$

where $\mathbf{u}_{\mathrm{r}}$ is the relative velocity between two fluids and is evaluated for each computation cell as follows:

$$\mathbf{u}_{\mathrm{r}} = \left(\min\left(C_{\mathrm{k}}\left|\mathbf{u}\right|, \max\left(\left|\mathbf{u}\right|\right)\right)\right)\frac{\nabla\alpha_{\mathrm{k}}}{\left|\nabla\alpha_{\mathrm{k}}\right|} \tag{5}$$

where C_{k} is the compression coefficient, determining the degree of compression.

$$\rho = \sum_{\mathrm{k}} (\alpha_{\mathrm{k}}\rho_{\mathrm{k}}) \tag{6}$$

$$\mu = \sum_{\mathrm{k}} (\alpha_{\mathrm{k}}\mu_{\mathrm{k}}) \tag{7}$$

$\mathbf{F}_{\mathrm{st}}$ is evaluated using the continuum surface force model as follows:

$$\mathbf{F}_{\mathrm{st}} = \gamma\kappa\nabla\alpha \text{ with } \kappa = -\nabla \cdot \mathbf{n} \tag{8}$$

where κ is mean curvature and $\mathbf{n}$ is unit normal vector of the interface (far from the wall), which is defined as follows:

$$\mathbf{n} = \nabla\alpha / \left|\nabla\alpha\right| \tag{9}$$

At solid surface, $\mathbf{n}$ is modified by contact angle.

The size of a computation cell is 19.25 μm cubed. Across the 300-μm thick overlayer, there are 15 cells. Across droplet radius there are approximately 57 cells.

Supplementary Note 2. Heat transfer of spaying cooling.

For the natural cooling, the temperature during cooling can be describe as follows[3]:

$$T(t) = T_0 + (T_{\text{initial}} - T_0)e^{-pt} \text{ with } p = h_{\text{natural}} A_{\text{al}} \big/ m_{\text{al}} c_{\text{p}}^{\text{al}} \tag{10}$$

where T_0, T_{initial} are ambient and initial aluminum temperature, respectively; h_{natural} is the convective heat transfer coefficient; A_{al}, m_{al}, c_{p}^{al} are the area, mass, and specific heat capacity of aluminum plate, respectively. In our case, h_{natural} = 15 W m^{-2} ^{o}C^{-1}, A_{al} = 0.02 m^2, m_{al} = 62.2 g, c_{p}^{al} = 0.9 J g^{-1} ^{o}C^{-1}. Given T_{initial} = 251 ^{o}C, the average cooling rate r_{cooling} for natural cooling is 1.43 ^{o}C s^{-1}, to which our experimental result (1.59±0.26 ^{o}C s^{-1}) matches.

The spraying water expedites the cooling through forced convection. The heat taken by the spraying water Q_{w} is as follows:

$$Q_{\text{w}} = Q_{\text{al}} - Q_{\text{natural}} \text{ with } Q_{\text{al}} = c_{\text{p}}^{\text{al}} m_{\text{al}} (T_{\text{initial}} - T_0) \tag{11}$$

where Q_{natural} is the heat loss due to natural cooling.

The heat capacity Q of water is as follows:

$$Q = Q_{\text{sensible}} + Q_{\text{latent}} \text{ with } Q_{\text{sensible}} = c_{\text{p}}^{\text{w}} m_{\text{w}} \Delta T_{\text{w}} \text{ ; } Q_{\text{latent}} = q_{\text{latent}} m_{\text{w}} \tag{12}$$

where Q_{sensible}, Q_{latent} are the sensible and latent heat capacity, respectively; c_{p}^{w}, m_{w}, q_{latent} are the specific heat capacity, mass, and specific latent heat of water, respectively; ΔT_{w} = 100 (^{o}C)-T_0.

The heat transfer efficiency is described by Q_{w}/Q. In our case, c_{p}^{w} = 4.2 J g^{-1} ^{o}C^{-1}, m_{w} = 2.0 g, q_{latent} = 2266 J g^{-1}, giving Q = 5170.4 J. Using the overlaid water droplets for cooling, we get Q_{w}/Q = 43.3 %. The volume ratio between silicone oil and water is 0.05, and the sensible heat of silicone oil is about 0.6% of that of water which has been neglected.

Supplementary Note 3. Lubricated sliding.

With sufficient lubricant, the inviscid water droplet oleoplans at a velocity U_s and entrains a subjacent lubricant film of microscale thickness h (Supplementary Fig. 9c, d). The viscous drag F_d associates with Landau-Levich-Derjaguin flow[4-6]. Droplet front meniscus can be divided into three regions: the static meniscus, the entrained lubricant film, and the dynamic meniscus connecting the two (Supplementary Fig. 9d). By matching the pressure at dynamic and static meniscus, the dynamic meniscus length can be obtained as $l \sim \sqrt{R_0 h/2}$. In the dynamic region, the surface tension counteracts the meniscus deformation caused by viscous stress. A balance between the two forces yields the lubricant film thickness as $h \sim R_0 \mathrm{Ca}^{2/3}$, where Ca is the capillary number $\mu_o U_s / \gamma_{d/o}$. As a result, we can have the viscous drag $F_d \sim \mu_o U_s l \pi D / h \sim \pi \gamma_{d/o} D \mathrm{Ca}^{2/3}$.

As shown in Supplementary Fig. 9e, once the depleted lubricant is insufficient to sustain the lubricated sliding, it is expected that the F_d increases substantially, a fact that is experimentally confirmed in Supplementary Fig. 9f. We define the lubricated sliding length L as the distance travelled by the droplet till F_d starts to increase (Supplementary Fig. 9f). Having the approximation of lubricant film thickness h, the sliding length L can be estimated as $L \sim V_o D^{-2} Ca^{-2/3}$ through mass conservation.

Supplementary Table 1. The tested droplet-overlayer combinations.

Droplet	Overlayer	*S*	Stability	Experimentally matched
Water	Silicone oil (0.6 mPa s)	6.7	Stable	Yes
Water	Silicone oil (4.6 mPa s)	2.7	Stable	Yes
Water	Silicone oil (20 mPa s)	2.3	Stable	Yes
Water	Silicone oil (100 mPa s)	2.1	Stable	Yes
Water	Hexane	3.3	Stable	Yes
Water	Perfluorohexane	4.2	Stable	Yes
Water	Ferrofluid	17.8	Stable	Yes
50% water glycerol mixture	Dodecane	32.3	Stable	Yes
10 M HCl	Dodecane	28.2	Stable	Yes
10 M NaOH	Dodecane	11.1	Stable	Yes
Hexadecane	Krytox VPF1506	2.9	Stable	Yes
Soybean oil	Krytox VPF1506	10.0	Stable	Yes

Supplementary Table 2. A list of the symbols.

θ	Apparent contact angle
D_0	Diameter of droplet
R_0	Radius of droplet
D	Contact length/diameter of droplet on the surface
D_{min}	Size of rebounding fluid neck
H	Out-of-plane height
c	Thickness of overlayer
L	Sliding length
A	Surface area
V	Volume
κ	Mean curvature
t	Time
t_i	Impact time
$t*$	Characteristic contact time
T	Temperature
T_0	Ambient temperature
$T_{initial}$	Initial temperature
$T_{boiling}$	Boiling temperature
$r_{cooling}$	Cooling rate
$h_{natural}$	Convective heat transfer coefficient
c_p	Specific heat capacity
$r_{cooling}$	Cooling rate
l	Specific latent heat
γ	Surface/interfacial tension

μ	Dynamic viscosity
ρ	Density
m	Mass
α	Phase fraction
C	Compression coefficient
$\mathbf{F}$	Force
F_{p}	Pinning force
F_{d}	Depinning/resistant force
f	Friction
p	Pressure
U_{i}	Impact velocity
U_{s}	Sliding velocity
$\mathbf{u}$, u	Flow velocity, and its magnitude
$\mathbf{g}$	Gravitational acceleration
q	Flow rate
E	Energy
W	Work
Q	Heat capacity
ϕ	Viscous dissipation rate per unit volume
We	Weber number
Ca	Capillary number
S	Spreading coefficient

Supplementary References

1 Chu, L. Y., Utada, A. S., Shah, R. K., Kim, J. W. & Weitz, D. A. Controllable monodisperse multiple emulsions. *Angew. Chem. Int. Ed.* **46**, 8970-8974 (2007).

2 Yeganehdoust, F., Attarzadeh, R., Karimfazli, I. & Dolatabadi, A. A numerical analysis of air entrapment during droplet impact on an immiscible liquid film. *Int. J. Multiphase Flow* **124**, 103175 (2020).

3 Bergman, T. L., Incropera, F. P., DeWitt, D. P. & Lavine, A. S. *Fundamentals of heat and mass transfer*. (John Wiley & Sons, 2011).

4 Landau, L. & Levich, B. in *Dynamics of curved fronts* 141-153 (Elsevier, 1988).

5 Daniel, D., Timonen, J. V., Li, R., Velling, S. J. & Aizenberg, J. Oleoplaning droplets on lubricated surfaces. *Nat. Phys.* **13**, 1020-1025 (2017).

6 Seiwert, J., Clanet, C. & Quéré, D. Coating of a textured solid. *J. Fluid Mech.* **669**, 55-63 (2011).
