# Supplementary material for: Slippery damper of an overlay for arresting and manipulating droplets on nonwetting surfaces
Source: Nat Commun. 2021 May 26;12:3154. doi: 10.1038/s41467-021-23511-3 (PMC8154893; doi:10.1038/s41467-021-23511-3)
Supplement: Supplementary file 3 — Description of Additional Supplementary Files [file 41467_2021_23511_MOESM3_ESM.pdf]

Description of Additional Supplementary Files

File name: Supplementary Movie 1.
Description: Generation of overlaid droplets using co-flow microfluidic device.

File name: Supplementary Movie 2.
Description: The impact of pure and overlaid water droplets on superhydrophobic surface.

File name: Supplementary Movie 3.
Description: The impact of pure and overlaid hexadecane droplets on superomphiphobic surface.

File name: Supplementary Movie 4.
Description: The impact of pure and overlaid water droplets on superheated surface.

File name: Supplementary Movie 5.
Description: The impact of pure and overlaid water droplets on macro-textured superhydrophobic surface.

File name: Supplementary Movie 6.
Description: The impact of pure and overlaid water droplets on curved superhydrophobic surface.

File name: Supplementary Movie 7.
Description: Droplet sliding length control.

File name: Supplementary Movie 8.
Description: Pure and overlaid water droplets impact a superhydrophobic slope.

File name: Supplementary Movie 9.
Description: Post-deposition liquid manipulation.
